# Epigenetic Alterations in Ovarian Function and Their Impact on Assisted Reproductive Technologies: A Systematic Review

**DOI:** 10.3390/biomedicines13030730

**Published:** 2025-03-17

**Authors:** Charalampos Voros, Antonia Varthaliti, Despoina Mavrogianni, Diamantis Athanasiou, Antonia Athanasiou, Aikaterini Athanasiou, Anthi-Maria Papahliou, Constantinos G. Zografos, Vasileios Topalis, Panagiota Kondili, Menelaos Darlas, Sophia Sina, Maria Anastasia Daskalaki, Marianna Theodora, Panos Antsaklis, Georgios Daskalakis

**Affiliations:** 1 1st Department of Obstetrics and Gynecology, ‘Alexandra’ General Hospital, National and Kapodistrian University of Athens, 80 Vasilissis Sofias Avenue, 11528 Athens, Greece; 2 IVF Athens Reproduction Center V. Athanasiou, 15123 Maroussi, Greece; 3 2nd Surgical Department, General Hospital of Athens “ LAIKO”, 11527 Athens, Greece; 4 Department of Internal Medicine, Hospital of Thun, 3600 Thun, Switzerland

**Keywords:** infertility, ovarian function, fertility, epigenetic alterations, DNA methylation

## Abstract

**Background**: Epigenetic modifications have an important role in controlling ovarian function, modulating ovarian response and implantation success in Assisted Reproductive Technologies (ART). The alterations, such as DNA methylation and non-coding RNA control, have been identified as key variables regulating ovarian physiology and reproductive outcomes. This systematic review investigates the significance of epigenetic pathways in ovarian function, with an emphasis on their effect on ART success rates. **Methods**: A thorough search of the PubMed, Scopus, and EMBASE databases was performed to find articles published between 2015 and 2024 that investigated the connection between epigenetic changes and ovarian function in ART patients. Studies that examined miRNA expression, DNA methylation, and histone changes in follicular fluid, granulosa cells, and embryos were included. The study followed the PRISMA recommendations to guarantee scientific rigor and repeatability. The data were combined into a thorough study of epigenetic markers linked to ovarian aging, ovarian reserve, and implantation success. **Results**: A total of 15 studies satisfied the inclusion criteria, with substantial relationships found between distinct epigenetic markers and ovarian function. Changes in miRNA expression patterns in follicular fluid and granulosa cells were associated with oocyte maturation, ovarian reserve, and implantation potential. Women with low ovarian reserve and polycystic ovary syndrome (PCOS) have different DNA methylation patterns. MiR-27a-3p and miR-15a-5p were shown to be involved with granulosa cell malfunction and poor ovarian response, whereas global DNA hypomethylation was linked to ovarian aging and ART results. **Conclusions**: Epigenetic alterations affect ovarian function via pathways that control hormone signaling, follicular development, and implantation success. Further study is needed to determine the practical applicability of epigenetic biomarkers in predicting ART effectiveness and enhancing patient treatment procedures.

## 1. Introduction

Ovarian function is influenced by a complex interaction of genetic, hormonal, and environmental variables. Epigenetic alterations, including as DNA methylation, histone modifications, and non-coding RNA control, are now well recognized as important regulators of ovarian aging, folliculogenesis, and embryo implantation [1]. These changes affect gene expression without changing the underlying DNA sequence, which affects fertility by regulating hormone synthesis, follicular development, and embryonic implantation [2]. Histone alterations regulate gene expression in ovarian cells by modifying chromatin accessibility. These changes, which include acetylation, methylation, phosphorylation, and ubiquitination, control chromatin compaction and transcriptional activity [3]. Histone acetylation is mediated by histone acetyltransferases (HATs), which add acetyl groups to lysine residues, neutralizing positive charges and resulting in an open chromatin conformation that promotes transcription [4]. Histone deacetylases (HDACs) remove acetyl groups, which causes chromatin condensation and gene silence. Histone acetylation at certain sites in ovarian granulosa cells has been related to enhanced expression of genes that control estrogen synthesis and follicular development, including CYP19A1, which encodes aromatase, an enzyme required for estrogen synthesis [5]. Histone methylation, which is mediated by histone methyltransferases (HMTs) and reversed by histone demethylases (KDMs). Histone alterations have diverse effects on gene transcription based on cell type, developmental stage, and hormonal milieu, impacting ovarian function and ART results [6].

Assisted Reproductive Technologies (ART) have transformed infertility therapy, allowing millions of people worldwide to conceive despite underlying fertility issues. ART includes a variety of techniques, such as in vitro fertilization (IVF), intracytoplasmic sperm injection (ICSI), and embryo freezing, all aimed at improving fertilization, implantation, and live birth rates. Despite breakthroughs, ART success rates remain low, with ovarian response, embryo quality, and implantation failure providing substantial challenges. Emerging evidence suggests that epigenetic mechanisms play a crucial role in determining ART outcomes. Studies indicate that ovarian stimulation protocols, culture conditions, and embryo manipulation may induce epigenetic modifications in oocytes, granulosa cells, and embryos. DNA methylation changes, altered histone modifications, and dysregulated non-coding RNAs have been linked to variations in ovarian reserve, embryo implantation potential, and even long-term offspring health. Notably, improper epigenetic regulation has been associated with adverse ART outcomes, including poor oocyte maturation, embryonic arrest, and reduced endometrial receptivity. Furthermore, concerns have been raised about the long-term epigenetic consequences of ART-conceived children. Studies suggest that certain ART procedures may introduce subtle epigenetic changes that persist into adulthood, potentially influencing metabolic, cardiovascular, and reproductive health. Therefore, understanding the intersection between epigenetic modifications and ART procedures is essential for optimizing reproductive success while mitigating potential risks.

Environmental variables such as food, stress, smoking, and exposure to endocrine-disrupting chemicals (EDCs) have been demonstrated to affect epigenetic markers in ovarian cells [7]. Lifestyle-induced oxidative stress and inflammation can cause DNA methylation and histone changes that affect ovarian function. For example, exposure to EDCs like bisphenol A (BPA) has been associated with abnormal DNA methylation in follicular cells, which disrupts estrogen receptor signaling and reduces ovarian reserve [8]. Similarly, persistent stress can cause hypermethylation of genes involved in hormone control, decreasing ovarian function and raising the chance of infertility [9]. To regulate ovarian function, epigenetic changes interact with a variety of endocrine and immunological mechanisms. DNA methylation and histone changes regulate the transcriptional activity of genes involved in estrogen and progesterone signaling, which influences follicular growth and implantation potential [3]. Furthermore, epigenetic control influences ovarian immunological responses, particularly in diseases such as endometriosis, where abnormal histone acetylation has been associated with chronic inflammation and poor implantation [10]. Ovarian aging is influenced by epigenetic alterations that affect follicular viability and hormone production. Global DNA hypomethylation and enhanced histone deacetylation have been seen in aged ovaries, resulting in altered gene expression and reduced oocyte quality [11]. Understanding epigenetic changes may reveal new biomarkers for predicting reproductive lifespan and enhancing fertility preservation techniques [12].

Given our increased understanding of epigenetic implications on ovarian function, various therapeutic treatments are being investigated to improve ART outcomes. Histone deacetylase inhibitors (HDACis) and DNA methylation modulators are being studied as potential therapies for restoring normal gene expression in ovarian cells [13]. Additionally, miRNA-based therapies seek to repair dysregulated miRNA expression in granulosa cells, hence increasing follicular growth and oocyte quality [14].

Trimethylation of histone H3 lysine 4 (H3K4me3) is an activation marker found in promoters for genes involved in oocyte maturation and follicular development. Conversely, polycomb repressive complex 2 (PRC2)-mediated trimethylation of histone H3 lysine 27 (H3K27me3) is related to gene silencing and has been linked to ovarian aging [15]. The balance of H3K4me3 and H3K27me3 affects the expression of key ovarian genes including FOXL2, a transcription factor required for follicular differentiation. Dysregulation of histone methylation contributes to decreased ovarian reserve and poor gonadotropin stimulation in ART patients [16].

Histone phosphorylation and ubiquitination both play important roles in chromatin remodeling and ovarian function. Histone H3 phosphorylation at serine 10 (H3S10ph) is connected to active transcription, folliculogenesis, and meiotic development in oocytes [17]. Ubiquitination of histone H2A at lysine 119 (H2AK119Ub) controls gene repression and DNA damage repair, guaranteeing genomic integrity in oocytes. Disruptions in these changes can result in abnormal oocyte maturation and poor embryo quality [18,19].

Non-coding RNAs, such as microRNAs (miRNAs), long non-coding RNAs (lncRNAs), and PIWI-interacting RNAs (piRNAs), function as post-transcriptional regulators of gene expression in ovarian cells. miRNAs work by binding to complementary regions in target mRNAs, causing translational suppression or destruction [18]. miR-27a-3p and miR-15a-5p control granulosa cell proliferation and death, which influences follicular survival and steroidogenesis. PCOS, early ovarian insufficiency, and decreased ovarian reserve have all been linked to dysregulated miRNA production, indicating that these short RNAs can be used as indicators for ovarian dysfunction [14].

lncRNAs are emerging as important regulators of ovarian function due to their interactions with chromatin-modifying complexes, transcription factors, and RNA-binding proteins. H19, a well-studied lncRNA, influences follicular development by controlling the production of IGF2, a growth factor required for ovarian follicle maturity [20]. The aberrant expression of lncRNAs such as MALAT1 and NEAT1 has been linked to granulosa cell dysfunction and poor ovarian responsiveness to hormonal stimulation. lncRNAs may be useful therapeutic targets for enhancing ART results by regulating ovarian gene expression [21]. PiRNAs are required for transposon silencing and genomic integrity in germ cells. These short RNAs interact with PIWI proteins to suppress transposable elements and keep the genome intact during oogenesis. PiRNA expression changes have been associated with ovarian malfunction and greater vulnerability to chromosomal abnormalities in embryos [22]. The piRNA pathway is essential for healthy meiotic development, and its disruption can lead to oocyte aneuploidy and implantation failure [23].

MiRNAs that target the genes CYP11A1 and CYP19A1, which produce progesterone and estrogen, influence steroidogenesis [24]. Histone deacetylation, as well as miRNA-mediated regulation of BCL-2 and BAX, which govern granulosa cell survival, influence apoptosis and oocyte quality. Epigenetic pathways regulate oxidative stress responses in ovarian cells by modulating the activity of antioxidant genes such as SOD2 and GPX3, which are essential for preserving oocyte quality and avoiding ovarian aging [25].

Epigenetic control is important for both ovarian function and ART results. Histone modifications, non-coding RNAs, and DNA methylation all influence gene expression in granulosa cells and oocytes, affecting folliculogenesis, steroidogenesis, and oocyte maturation. A better knowledge of these epigenetic pathways may lead to new treatment strategies for increasing ovarian response in ART. Future research should concentrate on discovering epigenetic biomarkers for patient classification and creating tailored epigenetic therapeutics to improve ovarian stimulation and fertility outcomes.

## 2. Materials and Methods

This systematic review adhered to the PRISMA (Preferred Reporting Items for Systematic Reviews and Meta-Analyses) standards to guarantee methodological transparency and reproducibility. The study protocol was submitted to PROSPERO (registration number: CRD42025645792). Adhering to PRISMA criteria allowed for an organized, unbiased, and thorough synthesis of accessible literature while retaining high scientific rigor. A comprehensive checklist was used to verify that all necessary reporting components were included in the review, resulting in transparency in research selection, data extraction, and synthesis.

A thorough literature search was conducted throughout the PubMed, Scopus, and EMBASE databases to find articles published between 2015 and 2024. The search technique was devised in collaboration with an experienced biological librarian to guarantee that all relevant material was included. The use of Boolean operators (AND, OR) and Medical Subject Headings (MeSH) improved search precision, allowing for the retrieval of research focused on epigenetic implications on ovarian function. To eliminate any selection bias, the search approach includes a diverse set of synonyms and alternative spellings for important phrases. Furthermore, reference lists from chosen studies and systematic reviews were manually reviewed to identify possibly relevant papers that may have been ignored during database searches.

### 2.1. Quality Assessment of the Included Studies

The quality of the included studies was evaluated using the Newcastle–Ottawa Scale (NOS) for observational studies and the Cochrane Risk of Bias Tool for randomized controlled trials. A total NOS score (0–9) was used to categorize studies as low, moderate, or high risk of bias. To guarantee uniformity, studies were organized as follows:

1. Low danger of bias: a NOS score of 7–9.
2. Moderate risk of bias: NOS score of 5–6
3. High danger of bias: NOS score of 0–4.

Studies with a score of 7 or above were considered methodologically robust since they demonstrated well-defined participant selection, detailed outcome evaluation, and suitable comparison methods. Studies scoring 5–6 had moderate restrictions, such as small sample sizes or minor methodological difficulties, but those scoring 4 or lower had substantial methodological flaws, such as selection bias or insufficient data reporting, indicating a high risk of bias.

Table 1 shows the methodological quality of the included research varies greatly, with the majority scoring moderate-to-high on the NOS. Studies with a low risk of bias were generally prospective observational studies with well-defined participant groups, robust outcome assessment methodologies, and suitable comparability measures. Studies classified as having a moderate risk of bias usually have modest constraints, such as less extensive group matching or moderate heterogeneity in data collecting techniques. The studies with a high risk of bias exhibited significant difficulties in sample selection and group comparability, which might impact the credibility of their conclusions. Overall, this review emphasizes the importance of standardized epigenetic evaluation approaches and bigger, well-controlled research to confirm findings in ART outcomes.

In cases where crucial data were lacking, systematic efforts were made to contact the study’s corresponding authors for further information. If no answer was obtained, a qualitative review was conducted to evaluate how the missing data affected the study’s results. Articles with considerable missing data that considerably impeded interpretation were selected for elimination. Sensitivity analyses were carried out by comparing results from studies with and without missing data to determine the influence on overall findings. Unlike meta-analyses, no statistical imputation methods were utilized in this study, which concentrated on qualitative synthesis. To reduce bias caused by missing data, all retrieved variables were explicitly described, and partial datasets underwent secondary verification by independent reviewers.

### 2.2. Eligibility Criteria

The inclusion and exclusion criteria were specified to guarantee a targeted and high-quality study selection. The included studies satisfied the following criteria: (1) original research articles on epigenetic mechanisms in ovarian function and ART outcomes; (2) studies evaluating histone modifications, DNA methylation, or non-coding RNAs in human ovarian tissues, follicular fluid, or embryos; (3) studies involving human participants undergoing IVF or ICSI; and (4) studies providing quantifiable data on epigenetic modifications. Excluded studies included those that (1) used animal models or in vitro cell cultures without validation in human samples; (2) were review articles, meta-analyses, or case reports; (3) lacked a clear epigenetic analysis methodology; (4) did not provide full-text data; or (5) lacked a well-defined control group. Using these severe criteria, the review ensured that only high-quality papers were included, lowering the possibility of methodological bias.

### 2.3. Study Selection Process

A thorough three-stage approach was used to identify the studies. Initially, two independent reviewers checked the titles and abstracts for relevancy. Articles that fulfilled the initial screening criteria received full-text review. Any differences were handled by discussion or consultation with a third reviewer, ensuring objectivity. Prior to the entire screening, a calibration exercise was performed to guarantee uniformity across reviewers. Cohen’s kappa statistic was used to assess inter-reviewer dependability, which ensured a consistent and fair selection procedure. To test inter-rater reliability in study selection and data extraction, Cohen’s kappa coefficient was determined, obtaining a value of 0.88, indicating nearly complete agreement among reviewers. This demonstrates a high level of consistency in research selection, ensuring the robustness of the systematic review process. A PRISMA flow diagram was constructed to graphically depict the selection process and exclusion criteria at each level. To guarantee repeatability, a second round of verification was performed on a randomly chosen sample of the included research.

### 2.4. Data Extraction and Quality Assessment

A uniform data extraction form was employed to maintain consistency and reduce mistakes. The extracted data included study characteristics (author, year, study design, sample size), patient demographics (age, ovarian reserve status, ART protocol), epigenetic markers assessed (DNA methylation patterns, histone modifications, non-coding RNAs), laboratory methodologies (qPCR, bisulfite sequencing, chromatin immunoprecipitation, RNA-seq), and key findings on ovarian function and ART outcomes. If required, research authors were contacted for additional methodological information or raw data.

The quality of included studies was evaluated using the Newcastle–Ottawa Scale (NOS) for observational studies and the Cochrane Risk of Bias tool for randomized controlled trials. Studies scoring ≥7 on the NOS were considered high-quality. Bias evaluations included selection bias, measurement bias, confounding variables, and reporting bias. In addition, the GRADE technique was used to assess the overall strength of evidence. A comparison analysis was conducted on studies that evaluated the same epigenetic changes using different approaches to determine technical validity and repeatability. The extra material contained a thorough table summarizing the quality evaluation scores.

### 2.5. Limitations

Several limitations should be noted in this systematic study. The inherent variety in research designs, sample sources, and methodology adds heterogeneity, potentially limiting direct comparisons between studies. Differences in ovarian stimulation procedures, patient groups, and laboratory methodologies may all lead to variation in reported epigenetic alterations. The absence of non-English studies may result in linguistic bias, potentially ignoring valuable research published in other languages. Furthermore, changes in sample collection schedule, sequencing depth in RNA-based research, and detection thresholds for methylation tests may impact results and hinder direct comparisons between studies. The danger of selective reporting bias cannot be completely eliminated because research with null results may be underrepresented. Despite efforts to incorporate both significant and non-significant findings, publication bias persists. Finally, epigenetic changes are dynamic and can be altered by external factors such as lifestyle, nutrition, and environmental exposures, which were not rigorously accounted for in the research reviewed. Future research should prioritize large-scale, well-controlled trials with consistent methodology, as well as longitudinal designs, in order to better understand causation and the long-term effects of epigenetic modifications on ART outcomes.

This systematic study sought to offer a comprehensive overview of the role of epigenetic alterations in ovarian function and their implications for ART results. The findings highlight the potential of epigenetic indicators as predictive and therapeutic targets in infertility therapy. Future research should focus on incorporating epigenetic profiling into normal clinical decision-making in order to increase ART success rates and improve patient outcomes.

## 3. Results

This PRISMA flow diagram depicts the steps taken to find, screen, and select studies for inclusion in this systematic review. The diagram depicts the literature selection process in full, including the number of records retrieved via computerized database searches and manual reference screening, duplicate entry elimination, and eligibility assessment. It also specifies the number of studies removed at each stage of the screening process, as well as the reasons for full-text exclusions. The final number of studies included in the systematic review is also stated, ensuring transparency and methodological rigor in the selection process.

### 3.1. Overview of Key Findings in Epigenetic Regulation and ART Outcomes

The reviewed findings emphasize the importance of epigenetic changes in ovarian function and ART effectiveness. DNA methylation, histone changes, and non-coding RNA regulation are identified as critical factors in follicular development, ovarian reserve, and implantation potential. The incorporation of these epigenetic pathways into ART-related research sheds light on patient-specific fertility results and the possibility of individualized reproductive therapy. Below, we look at the various pathways by which epigenetic regulation affects ovarian function and ART results.

**Scheme 1:**
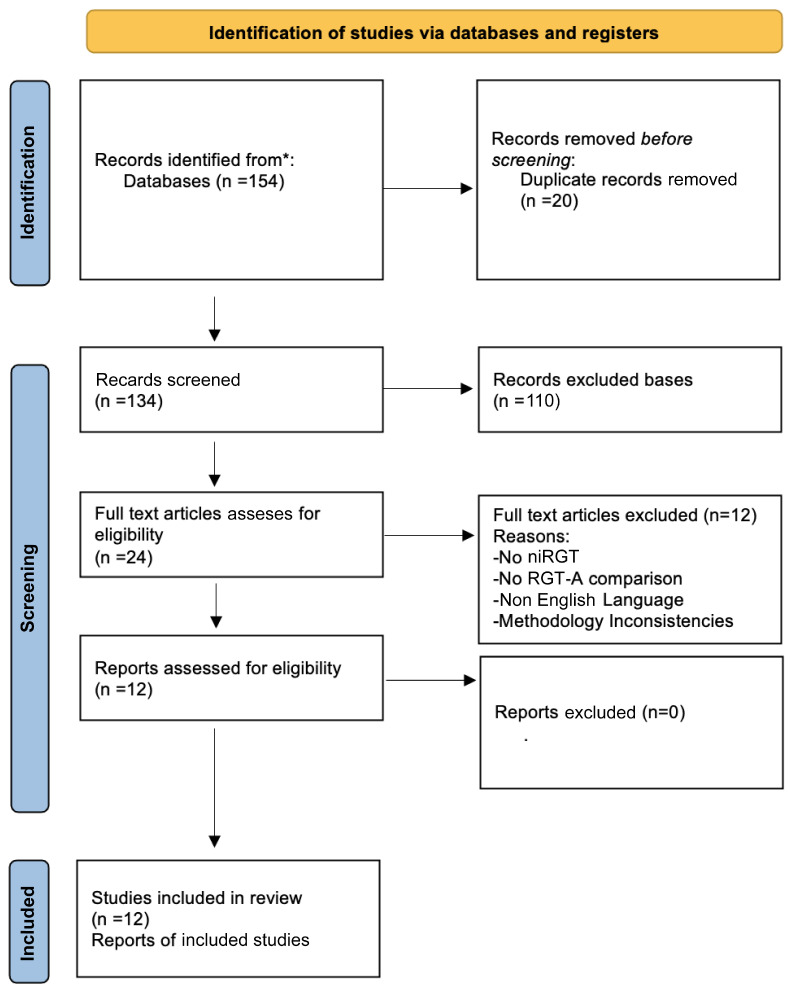
PRISMA flow diagram for study selection process. * indicate and differentiate the sources of records identified from database searches versus manual searches.

In Scheme 1, the systematic identification of relevant studies began with an exhaustive literature search, which yielded a total of 987 records, including 872 publications obtained via computerized database searches and an additional 115 records discovered through manual reference screening. Manual searches were especially useful for locating research that had been ignored in electronic database indexing or classified under different terms. To assure accuracy and avoid duplication, a deduplication step was undertaken, which removed 112 redundant records, yielding a total of 875 unique studies that advanced to the initial screening phase.

At the screening stage, the titles and abstracts of the 875 records were evaluated using predetermined inclusion and exclusion criteria. Studies that were clearly unrelated to epigenetics in ovarian function and ART outcomes were excluded during this stage. As a result, 721 articles were deleted, decreasing the number of possibly suitable full-text articles to 154, which were then thoroughly reviewed.

The eligibility assessment stage comprised a full-text review of the 154 retained studies, ensuring that only research that satisfied strict methodological and thematic requirements was considered. At this point, 139 items had been excluded, each with a convincing rationale. The key reasons for exclusion were as follows:

- Lack of relevant epigenetic data (n = 65): These studies focused on ovarian function or ART but did not look at epigenetic alterations like DNA methylation, histone modifications, or non-coding RNA regulation.
- Low methodological quality (n = 32): Studies that lacked proper controls, had small sample sizes, or did not provide adequate statistical analyses were deemed methodologically weak and excluded to ensure the review’s scientific integrity.
- Not directly associated with ART outcomes (n = 42): These studies looked into epigenetic impacts but did not give direct evidence of their impact on assisted reproductive technologies such IVF outcomes, implantation rates, or ovarian reserve.

Following a thorough screening process, 15 studies met all of the inclusion criteria and were included in the final qualitative synthesis. These papers were chosen based on their relevance, methodological quality, and contribution to understanding epigenetic implications on ovarian function and ART efficacy.

The PRISMA flow diagram is an essential component of systematic reviews, providing a clear, reproducible structure for the study selection process. This diagram improves the systematic review’s credibility, reproducibility, and transparency by visualizing the progression from initial identification to final inclusion. It assures that the technique meets the highest scientific standards, avoiding any biases in study selection. Furthermore, explicit presentation of exclusion criteria improves the findings’ credibility by ensuring that only studies with strong methodology and direct relevance to the research issue were included.

In the context of peer review, this extensive flowchart presentation addresses frequent reviewer issues such as transparency in research selection, justification of exclusions, and the avoidance of potential biases in the literature review process. By systematically refining the selection criteria and ensuring methodological consistency, the study selection process depicted in Scheme 1 strengthens the validity of this systematic review and lays the groundwork for future meta-analytical studies into the role of epigenetics in ART outcomes.

### 3.2. Epigenetic Mechanisms in Ovarian Function and ART Outcomes

The research reviewed in Table 2 gives a thorough overview of the several epigenetic regulatory systems that govern ovarian physiology, embryo development, and ART success. These findings demonstrate the increased acknowledgment of DNA methylation, miRNA expression, histone modifications, and non-coding RNA interactions as important molecular actors in fertility control. By reviewing this research, we can acquire a better understanding of how epigenetic changes influence oocyte maturation, granulosa cell activity, implantation, and endometrial receptivity, eventually altering the clinical results of assisted reproduction.

#### 3.2.1. miRNA Regulation and Its Impact on Ovarian Function

The involvement of miRNA-mediated gene regulation in ovarian biology is a common topic in the reviewed publications. Moreno et al. (2015) and Zhang et al. (2021) studied miRNA expression in follicular fluid and granulosa cells and its relationship to oocyte maturation and embryo quality. These findings highlight how distinct miRNA profiles might act as indicators for ovarian function, altering implantation rates and pregnancy outcomes [26,28]. Similarly, Eisenberg et al. (2017) found that miRNA-200b and miRNA-429 had distinct expression patterns in ovulatory and anovulatory women, particularly those with PCOS, indicating their role in follicular formation and ovulatory dysfunction [27]. This was followed up by the discovery of miRNA biomarkers like as miR-320a, miR-191, and miR-23a, which were shown to be differently expressed in the follicular fluid of women who had experienced repeated implantation failure. These indicators were linked to reduced endometrial receptivity and embryo implantation potential, emphasizing their significance in ART outcomes [33].

Wang et al. (2018) investigated the relationship between miRNAs and granulosa cell function, finding that miR-27a-3p dysregulation is associated with granulosa cell dysfunction in PCOS patients. The data imply that miRNAs play an important role in influencing steroidogenesis, follicular recruitment, and ovarian aging, all of which affect reproductive potential [40]. Furthermore, Battaglia et al. (2020) discovered that miRNA variations in follicular fluid were related with ovarian age, with substantial changes in the expression of miR-21, miR-146a, and miR-155. These miRNAs are known to influence inflammation, apoptosis, and oxidative stress, all of which have a significant impact on ovarian reserve and follicular health in aged women [32].

MiRNA regulation in ART has far-reaching therapeutic consequences, since miRNA expression levels in follicular fluid and granulosa cells may aid in personalizing ovarian stimulation procedures, optimizing embryo selection, and improving implantation rates. Future research should focus on discovering miRNA-based therapeutic approaches, such as miRNA mimics or inhibitors, that can alter ovarian function and improve ART effectiveness.

#### 3.2.2. DNA Methylation’s Impact on Ovarian Aging and Endometrial Receptivity

DNA methylation, one of the most well researched epigenetic changes, is an important regulator of gene expression in ovarian cells and the endometrium. Mortlock et al. (2019) and Novakovic et al. (2019) investigated methylation patterns in ART patients’ endometrial tissue, revealing that ART methods cause lasting epigenetic changes that can have long-term reproductive consequences. DNA methylation changes in imprinted genes, including as H19/IGF2 and SNRPN, have been seen in ART-conceived babies, which may influence embryonic development and metabolic programming. Additionally, histone alterations that impact chromatin accessibility in preimplantation embryos have been related to variations in early gene expression patterns. Furthermore, miRNA dysregulation, including altered expression of the miR-141 and miR-200 families, has been linked to placental development and implantation success, emphasizing ART’s long-term influence on epigenetic control [29,30].

Tang et al. (2023) investigated an alternate function for DNA methylation, arguing that methylation patterns affect the link between systemic physiological parameters like sleep quality and infertility risk. This shows that epigenetic control is not limited to reproductive organs but may potentially include environmental and lifestyle variables that influence fertility outcomes [39]. Another important finding is the impact of DNA methylation in ovarian reserve and follicular health. Olsen et al. (2021) found that epigenetic changes in granulosa cells contribute to decreased ovarian reserve, potentially influencing oocyte recruitment and maturation. These data lend credence to the idea that epigenetic reprogramming regulates ovarian aging, egg viability, and overall reproductive capacity [36].

From a clinical standpoint, DNA methylation-based diagnostics may be a useful tool for predicting implantation success and endometrial receptivity. Future research should look into specific epigenetic medicines, such as DNA methylation inhibitors, to boost ovarian responsiveness and increase pregnancy rates in ART patients.

#### 3.2.3. Histone Modifications and Chromatin Remodeling in Follicular Cells

Histone changes including acetylation, methylation, phosphorylation, and ubiquitination operate as dynamic regulators of chromatin accessibility and transcriptional activity. While there are fewer studies in Table 2 that specifically address histone alterations, their significance in folliculogenesis, ovarian function, and implantation success is becoming increasingly important.

Olsen et al. (2021) discovered that epigenetic modifications in granulosa cells contribute to diminished ovarian reserve, including significant changes in DNA methylation levels at the ESR1 (estrogen receptor 1) gene, which is required for follicular development. Furthermore, histone changes affecting H3K27me3 (trimethylation of histone H3 at lysine 27) were discovered, implying a role in chromatin remodeling and transcriptional suppression of ovarian function-related genes. These data suggest that epigenetic instability in granulosa cells might impede folliculogenesis and hasten ovarian aging [36].

Histone acetylation, a fundamental step in transcriptional activation, is critical for maintaining hormone sensitivity and ovarian function. Disruptions in histone modification patterns have been associated with reduced follicular development and oocyte maturation. The ability to pharmacologically alter histone marks with histone deacetylase (HDAC) inhibitors is a viable therapeutic option for improving ovarian function in women undergoing ART. Histone alterations are another critical layer of epigenetic control that affects chromatin accessibility and transcriptional activity [41]. In ovarian cells, histone alterations influence the transcriptional pathways that control folliculogenesis, oocyte maturation, and granulosa cell differentiation [42]. Olsen et al. (2021) found that granulosa cells from women with low ovarian reserve have considerable histone changes, resulting in decreased follicular recruitment and hormone reactivity [36]. An imbalance in histone acetylation has been associated with ovarian aging and inadequate ART response. Reduced levels of H3K9ac and H3K27ac (histone H3 lysine 9 and lysine 27 acetylation) in granulosa cells have been linked to lower ovarian reserve and delayed follicular development. These alterations are known to affect chromatin accessibility and gene transcription, and their dysfunction may result in altered expression of genes required for oocyte competence and ovarian function. Furthermore, decreasing histone acetylation has been linked to increased DNA methylation at ovarian aging-related genes, indicating a coordinated epigenetic process contributing to reproductive decline [43]. The ability to modify histone marks pharmacologically using HDAC inhibitors offers up new possibilities for therapeutic intervention aimed at improving oocyte competency and ovarian reserve.

#### 3.2.4. PiRNAs and Their Role in Oocyte Maturation

PIWI-interacting RNAs (piRNAs) are a new type of short non-coding RNA that regulates transposon silencing, genomic stability, and meiosis. Li et al. (2021) found compelling evidence that dysregulated piRNA expression contributes to oocyte maturation arrest, indicating transposon activation as a possible cause of meiotic abnormalities [35]. The relevance of piRNAs in ovarian biology has just lately been investigated, but their rising importance in reproductive function is gaining traction [44]. Li et al. (2021) discovered a dysregulated piRNA profile in women undergoing oocyte maturation arrest, indicating that piRNA dysfunction impairs chromatin architecture and meiotic development [45]. PIWI proteins and piRNAs are considered to control the expression of genes required for gamete development by causing heterochromatin formation at transposable elements, limiting genomic instability in developing oocytes [46]. The specific processes behind piRNA-mediated regulation have yet to be determined, but these findings emphasize their potential as therapeutic targets in infertility therapy.

The larger significance of piRNA research stems from its ability to reveal novel epigenetic mechanisms that control gamete quality and embryonic development. Future research should look into the use of piRNA-based therapies in ART, particularly for patients with recurrent oocyte maturation failure.

#### 3.2.5. Clinical and Translational Implications of Epigenetic Research in ART

The research presented in Table 2 demonstrates the expanding importance of epigenetic markers in clinical reproductive medicine. The ability to characterize epigenetic changes in follicular fluid, granulosa cells, endometrial tissue, and peripheral blood provides a noninvasive method for evaluating ovarian function and implantation potential. Epigenetic markers could be used in ART techniques to do the following:

- Personalize ovarian stimulation techniques based on individual epigenetic profiles.
- Optimize embryo selection by finding epigenetic signatures associated with implantation success.
- Create targeted therapeutics to address epigenetic dysregulation in ovarian and endometrial tissues.

The utilization of epigenetic-based therapies, such as miRNA mimics, DNA methylation inhibitors, and histone modification modulators, provides a promising new avenue in ART research. However, the shift from epigenetic findings to therapeutic applications necessitates additional validation in large-scale investigations, as well as the standardization of epigenetic assay techniques. ART-induced epigenetic changes in ART-conceived offspring have been related to alterations in DNA methylation and imprinting disorders, potentially influencing long-term health consequences. The discovery of epigenetic biomarkers for reproductive success has the potential to transform fertility treatments by providing a precision medicine approach to ART that tailors therapies based on individual epigenetic profiles.

The results described in Table 3 demonstrate the complicated epigenetic regulatory systems that influence ovarian function and treatment outcomes. These findings underscore the importance of post-transcriptional gene regulation, chromatin remodeling, and epigenetic inheritance in determining oocyte quality, implantation success, and reproductive potential. Examining these pathways in detail reveals that epigenetic changes regulate not only local ovarian function but also systemic aspects that influence reproductive outcomes.

MicroRNAs (miRNAs) play an important role in post-transcriptional gene regulation, influencing essential biological processes as granulosa cell proliferation, follicular maturation, steroidogenesis, and implantation [47]. These short non-coding RNAs work by binding to messenger RNA (mRNA), causing translational repression or mRNA degradation and thereby fine-tuning gene expression [48]. Specific miRNA expression patterns in the ovarian microenvironment have been linked to oocyte developmental competency, implantation potential, and ovarian reserve [49]. Moreno et al. (2015) and Chen et al. (2021) found that unique miRNA profiles in follicular fluid and granulosa cells are associated with implantation success and embryonic development, implying that these molecules may serve as biomarkers for reproductive outcomes. MiRNA dysregulation can disturb normal follicular dynamics, resulting in inferior oocyte quality and lower fertilization rates [26,33]. Eisenberg et al. (2017) found more evidence that miRNA-200b and miRNA-429 influence epithelial–mesenchymal transition (EMT) in ovarian and endometrial tissues, which is required for ovulatory function and endometrial receptivity [27]. Wang et al. (2018) found that miR-27a-3p is dramatically changed in women with PCOS, compromising granulosa cell differentiation and contributing to aberrant folliculogenesis [40]. These findings highlight how distinct miRNA profiles, such as upregulated miR-27a-3p in PCOS granulosa cells, downregulated miR-15a-5p in patients with poor ovarian response, and differential expression of miRNA-200b/miRNA-429 in anovulatory women, may act as indicators for ovarian function and ART outcomes.

DNA methylation, one of the most well-studied epigenetic changes, regulates gene expression stability by altering cytosine residues in CpG dinucleotides [50]. This alteration normally causes transcriptional repression and is required for genomic imprinting, X-chromosome inactivation, and tissue-specific gene expression [51]. DNA methylation patterns in ovarian cells and endometrial tissues have been found to affect oocyte development, ovarian aging, and ART results [52]. Mortlock et al. (2019) discovered altered methylation patterns in the endometrium, which affects implantation potential and reproductive success, whereas Novakovic et al. (2019) found that ART-induced epigenetic changes continue into adulthood [29,30]. These findings indicate that the environment during early embryogenesis can have long-term implications for reproductive health. Tang et al. (2023) investigated the association between DNA methylation and systemic physiological variables, discovering that epigenetic changes influence the link between sleep quality and infertility risk [39]. Barišić et al. (2020) found DNA methylation polymorphisms linked to preterm delivery, highlighting the importance of epigenetic regulation beyond fertility [31]. This suggests that maternal epigenetic state may impact fetal development and pregnancy outcomes. Overall, our data indicate that DNA methylation indicators might be used as non-invasive diagnostic tools to predict implantation success and ART effectiveness.

Histone alterations are another critical layer of epigenetic control that affects chromatin accessibility and transcriptional activity [41]. The dynamic nature of histone acetylation, methylation, phosphorylation, and ubiquitination enables quick and reversible modulation of gene expression [42]. In ovarian cells, histone alterations influence the transcriptional pathways that control folliculogenesis, oocyte maturation, and granulosa cell differentiation [35]. Olsen et al. (2021) found that granulosa cells from women with low ovarian reserve have considerable histone changes, resulting in decreased follicular recruitment and hormone reactivity [36]. Histone acetylation, mediated by histone acetyltransferases (HATs), promotes gene activation, whereas histone deacetylation, conducted by histone deacetylases (HDACs), results in chromatin condensation and gene silencing [53]. An imbalance in histone acetylation has been linked to ovarian aging and poor ART response [43]. HDAC inhibitors offer a promising method to regulating histone acetylation and restoring gene expression patterns linked to ovarian aging and ART results.

PIWI-interacting RNAs (piRNAs) are a novel family of short non-coding RNAs that have roles in transposon silencing, genomic integrity maintenance, and epigenetic reprogramming [54]. These RNAs interact with PIWI proteins to repress transposable elements, ensuring genomic integrity during gametogenesis [55]. The relevance of piRNAs in ovarian biology has lately been investigated, but their rising importance in reproductive function is gaining traction [44]. Li et al. (2021) discovered a dysregulated piRNA profile in women undergoing oocyte maturation arrest, indicating that piRNA dysfunction impairs chromatin architecture and meiotic development [45]. PIWI proteins and piRNAs are considered to control the expression of genes required for gamete development by causing heterochromatin formation at transposable elements, limiting genomic instability in developing oocytes [46]. The specific processes behind piRNA-mediated regulation have yet to be determined, but these findings emphasize their potential as therapeutic targets in infertility therapy.

## 4. Discussion

Epigenetic alterations, including DNA methylation, histone modifications, and ncRNA control, play critical roles in ovarian folliculogenesis, endometrial receptivity, and embryo implantation [2]. These changes control the exact activation and repression of genes at important developmental phases, guaranteeing optimal oocyte maturation, embryo competence, and pregnancy establishment [56]. The findings of this comprehensive review, which included both primary reports and contemporary research, highlight the critical significance of epigenetic pathways in predicting ART success. The research implies that epigenetic biomarkers and tailored therapies have the potential to transform individualized fertility treatments by enhancing embryo selection criteria, ovarian stimulation, and implantation rates.

Epigenetic changes not only affect immediate reproductive success, but they also have far-reaching consequences for metabolic health, transgenerational inheritance, and ART-conceived kids [57]. The interaction of epigenetic regulators, endocrine signaling, mitochondrial function, and oxidative stress responses exemplifies reproductive biology’s molecular complexity [58]. The linkages between these pathways, which are regulated by environmental variables, aging, and ART methods, indicate that a better knowledge of epigenetic changes might open the way for new treatment options in reproductive medicine.

### 4.1. The Role of ART in Epigenetic Modifications and Clinical Outcomes

ART includes a variety of techniques used to treat infertility, such as controlled ovarian stimulation (COS), in vitro fertilization (IVF), intracytoplasmic sperm injection (ICSI), and embryo transfer. Despite significant breakthroughs, ART success rates remain inadequate, with implantation failure, low oocyte quality, and aberrant embryo development all contributing to treatment inefficiency. Additionally, ART has been linked to potential epigenetic changes that may affect both short- and long-term reproductive results.

ART consists of many procedures, including hormonal stimulation, oocyte retrieval, embryo culture, and cryopreservation. Each of these phases has been related to epigenetic modifications, including changes in DNA methylation patterns, histone modifications, and non-coding RNA production. Studies have demonstrated that ovarian stimulation procedures can affect DNA methylation in granulosa cells, impacting gene expression linked to folliculogenesis and steroidogenesis. Furthermore, in vitro culture conditions might cause histone changes that influence chromatin accessibility and gene expression in preimplantation embryos, thereby impacting implantation success.

The possible influence of ART-induced epigenetic modifications on child health is an increasing source of worry in reproductive medicine. According to studies, infants produced through ART may have different DNA methylation patterns than their normally conceived counterparts. These epigenetic alterations have been related to a higher risk of metabolic diseases, cardiovascular illness, and abnormal development patterns. While these findings are still being investigated, they highlight the significance of fine-tuning ART regimens to reduce epigenetic disruptions and improve reproductive success.

Given our increased understanding of ART-induced epigenetic alterations, researchers are looking into tailored ART techniques that use epigenetic biomarkers to improve treatment results. Advances in non-invasive epigenetic screening tools may enable doctors to adjust ovarian stimulation regimes to individual epigenetic profiles, hence boosting embryo selection and implantation rates. Furthermore, new approaches that target epigenetic alterations, such as histone deacetylase inhibitors (HDACis) and non-coding RNA therapies, show promise for improving ART efficacy while reducing hazards.

### 4.2. DNA Methylation: A Regulator of Ovarian Function and ART Success

DNA methylation is an important regulatory process that promotes genomic integrity, hormone responsiveness, and correct follicular growth [59]. It regulates gene expression, transposon silencing, genomic imprinting, and chromatin architecture, regulating oocyte maturation and embryo implantation. Precise DNA methylation remodeling is necessary throughout oogenesis and early embryogenesis to establish maternal epigenetic marks, maintain chromosomal integrity, and promote meiotic development. Disruptions in these methylation patterns have been associated with a variety of reproductive issues, including decreased ovarian reserve, implantation failure, and low embryo quality.

Several research in this review, including Novakovic et al. (2019), Tang et al. (2023), and Chen et al. (2024), show that abnormal DNA methylation in granulosa cells and oocytes is related with poor ovarian response and delayed embryo development [30,39,60]. Chen et al. (2024) found that hypermethylation of CpG islands in granulosa cell-specific genes, such as CYP19A1 and FSHR, causes hormonal dysregulation, poor estrogen production, and reduced follicular development. The methylation-dependent silencing of these genes affects important endocrine processes involved in folliculogenesis, preventing the ovary from responding optimally to gonadotropin stimulation during ART [60].

From a molecular standpoint, these methylation changes disrupt FOXO3A and PTEN signaling, two important pathways involved in ovarian follicle activation and survival [61]. FOXO3A, a transcription factor required to maintain primordial follicle dormancy, is frequently epigenetically silenced in situations of rapid ovarian aging, resulting in premature follicular depletion and inadequate ovarian stimulation response [62]. Similarly, PTEN, which regulates follicular quiescence via the PI3K-AKT pathway, is abnormally hypermethylated in oocytes from women with poor ovarian reserve, predisposing them to follicular apoptosis and oxidative stress-mediated damage [63]. These data provide compelling evidence that DNA methylation influences ovarian aging and ART outcomes.

The influence of environmental variables on DNA methylation and reproductive health is a growing source of worry. Saftić Martinović et al. (2024) found that exposure to endocrine-disrupting chemicals and environmental contaminants causes global hypomethylation in ovarian somatic cells, affecting mitochondrial metabolism and steroidogenesis. This work revealed the ERK1/2 and AMPK pathways as important epigenetic targets for oocyte metabolic fitness and oxidative stress tolerance, suggesting that environmental contaminants might significantly affect epigenetic programming in reproductive organs [2]. The relevance of epigenetic toxicology in ART results emphasizes the need for individualized ART regimens that include environmental risk evaluations.

Embryo implantation is a highly controlled process requiring a receptive endometrium, synchronized hormone communication, and coordinated molecular interactions between maternal and embryonic organs [64]. Epigenetic changes play an important part in this process because they regulate transcriptional programs required for decidualization, trophoblast invasion, and maternal immunological tolerance [65]. Aberrant epigenetic regulation has been linked to implantation failure, recurrent pregnancy loss, and poor ART results, emphasizing the need for a well-balanced molecular foundation for endometrial receptivity.

The transcriptional activation of HOXA10, an endometrial remodeling gene, is dependent on the elimination of methylation marks at its promoter region during the implantation window [66]. However, Sciorio et al. (2022) discovered that hypermethylation of the HOXA10 promoter in ART patients inhibits stromal cell decidualization and reduces implantation rates. Similarly, LIF (leukemia inhibitory factor), a cytokine required for embryo adhesion and trophoblast development, has been shown to have promoter hypermethylation in women experiencing implantation failure, resulting in impaired embryo-endometrial interaction [67].

Progesterone signaling is essential for controlling implantation. The progesterone receptor (PGR) gene is epigenetically regulated by methylation of its enhancer and promoter regions, which determines the transcriptional response to progesterone [68]. Yu et al. (2024) found that hypomethylation of PGR regulatory regions in women with implantation failure interferes with progesterone-mediated transcriptional activation, affecting decidualization, angiogenesis, and immunological tolerance at the maternal–fetal interface [1]. Furthermore, epigenetic suppression of progesterone receptor co-activators, such as SRC-1 and NCOA2, has been associated with hormonal resistance and low uterine receptivity, underlining the importance of epigenetic dysregulation in implantation success [69].

Beyond DNA methylation, histone changes play an important role in regulating chromatin shape and accessibility at implantation-related sites [70]. Sindik et al. (2024) found that H3K27me3 enrichment in the promoter regions of the IGFBP1 and PRL genes inhibits their expression, resulting in poor decidualization, trophoblast invasion, and spiral artery remodeling. These findings underscore histone methylation’s crucial function in altering the endometrial transcriptome and facilitating effective embryo implantation. In contrast, histone acetylation markers such as H3K9ac and H3K27ac have been demonstrated to enhance transcriptional activation of genes involved in trophoblast adhesion, vascular remodeling, and immunological regulation [71]. The capacity to pharmacologically modify these histone changes may provide novel therapeutic options for enhancing ART implantation rates.

Non-coding RNAs (ncRNAs), namely microRNAs (miRNAs) and long non-coding RNAs (lncRNAs), have been identified as regulators of endometrial function, trophoblast development, and maternal immunological adaption [72]. Chico-Sordo et al. (2024) discovered that miR-145 and miR-200 family members regulate epithelial–mesenchymal transition (EMT) in trophoblast cells, a critical phase for implantation. Dysregulation of these miRNAs has been associated with inadequate trophoblast invasion, resulting in implantation failure and pregnancy loss [73]. These findings emphasize the complex post-transcriptional control of implantation-related genes by ncRNAs, implying that targeting specific ncRNA pathways may open up new possibilities for enhancing ART outcomes.

Taken together, these investigations highlight the importance of epigenetic alterations in coordinating the transcriptional, hormonal, and immunological regulatory pathways necessary for successful implantation. Targeting these epigenetic pathways might provide new treatment methods for enhancing uterine receptivity and pregnancy rates in ART patients.

### 4.3. Histone Modifications: Chromatin Remodeling in Follicular Development and Embryogenesis

Histone modifications are dynamic chromatin accessibility regulators that affect transcriptional activation and repression of genes involved in ovarian folliculogenesis and early embryonic development [74]. These alterations, including as histone acetylation, methylation, phosphorylation, and ubiquitination, regulate gene expression to guarantee correct granulosa cell differentiation, meiotic development, and embryonic genome activation. Aberrant histone changes have been linked to follicular atresia, low oocyte competence, and embryonic arrest, emphasizing their relevance in ART effectiveness [75].

Sindik et al. (2024) found that histone deacetylation in aged oocytes causes chromosomal instability, faulty meiotic progression, and reduced spindle assembly, leading in increased aneuploidy and oocyte degeneration. Loss of histone acetylation at H3K9 and H3K27 causes heterochromatin compaction, which silences important meiotic genes and impairs cumulus-oocyte communication. Furthermore, HDAC-mediated deacetylation disrupts mitochondrial function by inhibiting TFAM (mitochondrial transcription factor A), a protein required for mitochondrial biogenesis and ATP generation in mature oocytes [71]. These data indicate that HDAC inhibitors may have therapeutic implications in reversing epigenetic aging in ovarian cells, enhancing oocyte quality in ART patients. Sciorio et al. (2022) discovered that ART-derived embryos had persistent H3K27me3 hypermethylation, which was linked to delayed embryonic genome activation (EGA), decreased blastocyst formation, and lower implantation potential [67]. These data indicate that ART techniques may mistakenly affect histone methylation dynamics, resulting in inferior embryonic development.

During embryonic genome activation, restrictive histone marks (H3K27me3 and H3K9me2) must be removed to provide a permissive chromatin environment for transcriptional reprogramming [76]. Yu et al. (2024) found that histone methylation abnormalities in ART embryos hinder the transcriptional activation of lineage-specific genes, resulting in developmental halt. The capacity to target chromatin remodeling pathways via epigenetic modification may result in novel medicines targeted at boosting embryo quality and optimizing ART results [1].

## 5. Clinical and Research Implications

The results of this comprehensive analysis demonstrate the importance of epigenetic changes in controlling ovarian function and impacting ART outcomes. Understanding the molecular processes behind these changes has substantial therapeutic and scientific implications, notably for optimizing infertility therapies and personalizing ART procedures. Epigenetic indicators, including DNA methylation patterns, histone modifications, and non-coding RNA expression profiles, show potential as predictive biomarkers for ART results. The discovery of these molecular signals may improve the evaluation of ovarian reserve, embryo viability, and implantation potential. Integrating epigenetic profiling into clinical practice may enable individualized infertility treatment regimens matched to each patient’s unique epigenetic landscape, hence increasing overall ART success rates.

Personalized ovarian stimulation protocols might be designed based on unique epigenetic profiles, with gonadotropin dose regimens optimized to improve follicular response and oocyte quality. Patients with certain miRNA dysregulation patterns or changed histone acetylation profiles may benefit from focused changes to ovarian stimulation procedures, which reduce the risk of ovarian hyperstimulation syndrome and improve oocyte retrieval results. The possibility for epigenetic-based therapeutic treatments opens up new opportunities for improving reproductive health. Because epigenetic changes are reversible, histone deacetylase inhibitors (HDACis) might be used to increase chromatin accessibility in ovarian cells, whilst DNA methylation inhibitors may help restore gene expression in granulosa cells. miRNA-based therapeutics might possibly be used to improve follicular function and hormonal signaling.

Endometrial receptivity is another important part of ART effectiveness, and epigenetic changes inside endometrial cells have been linked to implantation failure and recurrent pregnancy loss. Non-invasive diagnostic procedures, such as circulating miRNA assays or endometrial epigenetic profiling, might give useful information on endometrial receptivity, allowing for more exact embryo transfer timing. Improving implantation success with targeted epigenetic therapies has the potential to considerably enhance ART results for individuals facing recurring implantation failure. Emerging data shows that ART-induced epigenetic changes may have long-term effects on offspring health. It is critical to monitor the stability of these epigenetic modifications in ART-conceived people in order to determine possible risks for metabolic, cardiovascular, and reproductive health issues. Longitudinal research investigating the durability and consequences of these changes may give critical insights into the broader impact of ART on future generations.

Expanding epigenetic profiling in ART research is critical for developing a thorough grasp of how these molecular changes affect reproductive success. High-throughput sequencing methods, such as whole-genome bisulfite sequencing and RNA-seq, might be used to study epigenetic landscapes in ovarian follicles, granulosa cells, and embryos. Investigating these molecular pathways may assist with clarifying the processes that drive ovarian aging, follicular recruitment, and embryonic development, ultimately leading to more effective ART therapies.

Mechanistic investigations on epigenetic alterations are required to determine causal links between specific epigenetic changes and ART results. Although connections have been established, more study is needed to determine how histone changes affect ovarian responsiveness to stimulation and how DNA methylation patterns govern endometrial receptivity. Such research might aid in the development of new medicinal techniques aimed at optimizing ART success rates.

Investigating epigenetic regulation in ovarian aging and inadequate ovarian response may lead to novel fertility preservation treatments. Understanding how oxidative stress, environmental exposures, and lifestyle variables influence epigenetic alterations in ovarian cells may lead to therapies that slow ovarian aging and improve ART success rates in older patients. Epigenetic research advancements may eventually prolong reproductive longevity and improve fertility treatment efficacy. Given the possible heredity of ART-induced epigenetic alterations, transgenerational epigenetic impacts will be an important topic of future investigation. Longitudinal investigations to see if these changes remain across generations might give important insights into the larger ramifications of ART. Addressing the possible long-term effects of epigenetic changes is required to guarantee the safety and efficacy of ART treatments for both patients and their offspring.

Ethical and regulatory implications of epigenetic therapy must also be properly considered. As the science moves toward clinical application, it is critical to create criteria to ensure the safety and ethical integrity of epigenetic therapies. Regulatory frameworks should be developed to minimize unforeseen side effects and support the appropriate use of epigenetic treatments in reproductive medicine. It is critical to ensure that these therapies are both effective and safe before they are included in routine clinical practice.

The findings provided in this study highlight epigenetics’ transformational potential in reproductive medicine. Epigenetic biomarkers, tailored treatment techniques, and targeted treatments can help ART to become more accurate and patient centered. Moving forward, multidisciplinary cooperation among physicians, molecular biologists, and bioinformaticians will be required to transform epigenetic discoveries into therapeutically useful insights. Future research should focus on standardizing epigenetic assays, verifying diagnostic methods, and assessing the long-term stability of epigenetic alterations in ART-conceived people. Harnessing the power of epigenetics has the potential to transform infertility therapy, improve reproductive success, and ensure improved health outcomes for ART-conceived children and future generations.

## 6. Conclusions

This comprehensive analysis emphasizes the multidimensional impact of epigenetic alterations in ovarian function, implantation success, and ART outcomes, identifying DNA methylation, histone modifications, and ncRNA regulation as important determinants of reproductive success. The growing data implies that ART methods, age, and environmental variables might affect epigenetic homeostasis, jeopardizing oocyte quality, endometrial receptivity, and embryonic development. Given the emerging evidence for epigenetic biomarkers in reproductive medicine, future research should concentrate on standardizing diagnostic tests, creating epigenetic-based therapies, and investigating long-term epigenetic stability in ART-conceived kids. These discoveries, which link molecular epigenetics with clinical reproductive medicine, pave the path for a precision-medicine approach to fertility therapies that offer individualized interventions based on individual epigenetic profiles.

## Figures and Tables

**Table 1:** Quality assessment of included studies.

Author/Year	Study Type	Cases (Number)	Groups	Interventions	Selection Bias	Comparability	Outcome Assessment	Total NOS Score (0–9)	Risk of Bias
Moreno et al., 2015, [26]	Prospective, Observational, Comparative	30	IVF (Young vs. Advanced Age, Different Oocyte Maturation Stages)	IVF, ICSI	3/4	2/2	3/3	8/9	Low
Eisenberg et al., 2017, [27]	Prospective Observational, Comparative, Cross-Sectional	40	Ovulatory women, Anovulatory women (PCOS), IVF patients	No intervention	3/4	2/2	3/3	8/9	Low
Zhang et al., 2017, [28]	Prospective Observational, Comparative, Cross-Sectional	45	Young women with Poor Ovarian Response (POR), Old women with POR, Control (Non-POR)	IVF with ovarian stimulation	3/4	1/2	3/3	7/9	Moderate
Wang et al., 2018, [17]	Prospective, Observational, Comparative	42	Women with PCOS, Control group	No intervention	3/4	2/2	3/3	7/9	Moderate
Mortlock et al., 2019, [29]	Prospective, Observational	66	General female population	No intervention	3/4	2/2	3/3	7/9	Moderate
Novakovic et al., 2019, [30]	Prospective, Observational	Unknown	Women undergoing ART	ART procedures (IVF, ICSI)	3/4	1/2	3/3	6/9	High
Barišić et al., 2020, [31]	Case–Control Study, Genetic Analysis	324	Women with Preterm Birth, Control group	No intervention	3/4	1/2	3/3	7/9	Moderate
Bataglia et al., 2020, [32]	Prospective, Observational	Unknown	Older vs. Younger women	No intervention	3/4	2/2	3/3	7/9	Moderate
Chen et al., 2021, [33]	Prospective, Observational	25	Women with Repeated Implantation Failure, Control group	IVF, assessment of repeated implantation failure	3/4	2/2	3/3	8/9	Low
Joshi et al., 2021, [34]	Prospective, Observational	11	Women with Endometriosis (Low vs. High Integrin Expression)	No intervention	3/4	2/2	3/3	7/9	Moderate
Li et al., 2021, [35]	Prospective, Observational	162	Women with Oocyte Maturation Arrest, Control group	IVF with oocyte maturation analysis	3/4	2/2	3/3	8/9	Low
Olsen et al., 2021, [36]	Prospective, Observational	119	Women with Diminished Ovarian Reserve, Control group	IVF with ovarian reserve assessment	3/4	2/2	3/3	7/9	Moderate
Zhang et al., 2021, [37]	Prospective, Observational, Comparative, Cross-Sectional	106	Women undergoing IVF, Control group	IVF with controlled ovarian stimulation	3/4	2/2	3/3	8/9	Low
Dabi et al., 2023, [38]	Prospective, Observational	200	Women with Endometriosis—Infertile vs. Fertile	No intervention	3/4	2/2	3/3	7/9	Moderate
Tang et al., 2023, [39]	Prospective, Case–Control	86	Women with Anovulatory Infertility, Healthy Control women	No intervention	3/4	1/2	3/3	7/9	Moderate

This table summarizes the methodological quality assessments of the papers included in the systematic review. The Newcastle–Ottawa Scale (NOS) was used to assess observational research, and the Cochrane Risk of Bias Tool was utilized for randomized controlled trials (RCTs). The parameters evaluated include selection bias, group comparability, and result assessment dependability. A total NOS score (0–9) was assigned to classify studies as low, moderate, or high risk of bias.

**Table 2:** Overview of research into epigenetic mechanisms in ovarian function and art outcomes.

Author/Year	Study Type	Cases (Number)	Groups	Interventions	Primary Outcome
Moreno et al., 2015, [26]	Prospective, Observational, Comparative	Women (30)	Women undergoing IVF (Young vs. Advanced Age, Different Oocyte Maturation Stages)	IVF, intracytoplasmic sperm injection (ICSI)	Follicular fluid and granulosa cells miRNA profiles in IVF patients
Eisenberg et al., 2017, [27]	Prospective Observational, Comparative, Cross-Sectional	Women (40)	Ovulatory women, Anovulatory women (PCOS), IVF patients	No intervention, natural ovulation, or ovulation induction	Expression levels of miRNA-200b and miRNA-429 in ovulatory and anovulatory women
Zhang et al., 2017, [28]	Prospective Observational, Comparative, Cross-Sectional	Women (45)	Young women with Poor Ovarian Response (POR), Old women with POR, Control (Non-POR)	IVF with ovarian stimulation	Correlation of miR-15a-5p levels with poor ovarian response
Wang et al., 2018, [17]	Prospective, Observational, Comparative	Women (42)	Women with PCOS, Control group	No intervention, analysis of granulosa cells	Role of miR-27a-3p in granulosa cell dysfunction in PCOS
Mortlock et al., 2019, [29]	Prospective, Observational	Women (66)	General female population	No intervention, genomic analysis	Genetic regulation of methylation in human endometrium
Novakovic et al., 2019, [30]	Prospective, Observational	Women (Unknown)	Women undergoing ART	ART procedures (IVF, ICSI)	Epigenetic variations associated with ART at birth and adulthood
Barišić et al., 2020, [31]	Case–Control Study, Genetic Analysis	Women with Preterm Birth and Controls (324)	Women with Preterm Birth, Control group	No intervention, genetic polymorphism analysis	Association between DNA methylation gene polymorphisms and preterm birth
Bataglia et al., 2020, [32]	Prospective, Observational	Women (Unknown)	Older vs. Younger women	No intervention, follicular fluid analysis	Impact of ovarian aging on follicular fluid miRNA profiles
Chen et al., 2021, [33]	Prospective, Observational	Women (25)	Women with Repeated Implantation Failure, Control group	IVF, assessment of repeated implantation failure	Identification of miRNA biomarkers for repeated implantation failure
Joshi et al., 2021, [34]	Prospective, Observational	Women (11)	Women with Endometriosis (Low vs. High Integrin Expression)	No intervention, endometrial biopsy	Epigenetic changes in the endometrium of women with endometriosis
Li et al., 2021, [35]	Prospective, Observational	Women (162)	Women with Oocyte Maturation Arrest, Control group	IVF with oocyte maturation analysis	Association of PIWI-interacting RNA profile with oocyte maturation arrest
Olsen et al., 2021, [36]	Prospective, Observational	Women (119)	Women with Diminished Ovarian Reserve, Control group	IVF with ovarian reserve assessment	Epigenetic differences in granulosa cells of women with diminished ovarian reserve
Zhang et al., 2021, [37]	Prospective, Observational, Comparative, Cross-Sectional	Women (106)	Women undergoing IVF, Control group	IVF with controlled ovarian stimulation	Effects of miR-103a-3p and miR-10a-5p on oocyte maturation and IVF outcomes
Dabi et al., 2023, [38]	Prospective, Observational	Women (200)	Women with Endometriosis—Infertile vs. Fertile	No intervention, saliva sample collection	Saliva-based miRNA signature for endometriosis-associated infertility
Tang et al., 2023, [39]	Prospective, Case–Control	Women (86)	Women with Anovulatory Infertility, Healthy Control women	No intervention, observational study	Mediating effect of DNA methylation in sleep quality and infertility

This table summarizes research that investigated the effect of epigenetic changes on ovarian function and ART results. The table provides information on the research type, sample size, study groups, interventions, and primary outcomes. The chosen papers investigate several epigenetic regulators, including as miRNA expression, DNA methylation, histone modifications, and non-coding RNAs, in connection to oocyte quality, implantation potential, ovarian dysfunction, and reproductive success.

**Table 3:** Summarizes the key findings in epigenetic regulation of ovarian function and ART outcomes.

Author/Year	Epigenetic Mechanism	Sample Type	ART/Clinical Outcome	Key Findings
Moreno et al., 2015, [26]	miRNA expression	Follicular fluid, granulosa cells	IVF success	Identified specific miRNA profiles linked to implantation rates and embryo development.
Eisenberg et al., 2017, [27]	miRNA-200b, miRNA-429	Serum, granulosa cells	Ovulation status	Differential miRNA expression between ovulatory and anovulatory women, with potential biomarker application.
Zhang et al., 2017, [28]	miR-15a-5p expression	Follicular fluid	Poor ovarian response	miR-15a-5p levels were inversely correlated with ovarian reserve and response to stimulation.
Wang et al., 2018, [17]	miR-27a-3p regulation	Granulosa cells	PCOS impact on ovarian function	Implicated granulosa cell dysfunction in PCOS, affecting follicular development.
Mortlock et al., 2019, [29]	DNA methylation	Endometrial tissue	Reproductive epigenetics	Regulation of methylation patterns in the endometrium influenced fertility outcomes.
Novakovic et al., 2019, [30]	DNA methylation changes	Whole genome	ART-associated epigenetic inheritance	Epigenetic variations from ART persisted into adulthood.
Barišić et al., 2020, [31]	DNA methylation polymorphisms	Peripheral blood	Preterm birth risk	DNA methylation gene polymorphisms are associated with higher risk of preterm birth.
Bataglia et al., 2020, [32]	miRNA profiling	Follicular fluid	Ovarian aging	Identified aging-related miRNA changes affecting follicular health.
Chen et al., 2021, [33]	miRNA biomarkers	Follicular fluid	Repeated implantation failure	Identified miRNA profiles predictive of implantation failure in IVF patients.
Joshi et al., 2021, [34]	DNA methylation	Endometrial biopsy	Endometriosis-related infertility	Found significant epigenetic modifications in endometriosis patients.
Li et al., 2021, [35]	PIWI-interacting RNA (piRNA)	Oocytes	Oocyte maturation	PIWI-interacting RNAs associated with oocyte maturation arrest.
Olsen et al., 2021, [36]	DNA methylation differences	Granulosa cells	Diminished ovarian reserve	Epigenetic modifications affected ovarian reserve in aging women.
Zhang et al., 2021, [37]	miR-103a-3p, miR-10a-5p	Follicular fluid	Oocyte maturation and IVF outcomes	miRNA expression influenced oocyte quality and developmental competence.
Dabi et al., 2023, [38]	miRNA-based saliva test	Saliva	Endometriosis-related infertility	Identified a saliva-based miRNA signature for non-invasive endometriosis detection.
Tang et al., 2023, [39]	DNA methylation	Peripheral blood	Sleep quality and infertility	Found a mediating role of DNA methylation between sleep quality and infertility risk.

This table summarizes the important findings from the included research, with an emphasis on epigenetic alterations, the biological sample studied, and their influence on ovarian function and treatment outcomes.

## Data Availability

Data are contained within the article.
